# NONO Protein Regulates the Immune Response in Human Triple-Negative Breast Cancer Cells

**DOI:** 10.3390/ijms26178542

**Published:** 2025-09-02

**Authors:** Carmelina Antonella Iannuzzi, Iris Maria Forte, Marianna Tomeo, Anna Sfera, Francesco Pagano, Riziero Esposito Abate, Michelino De Laurentiis, Antonio Giordano, Luigi Alfano

**Affiliations:** 1 Department of Breast and Thoracic Oncology, Istituto Nazionale Tumori-IRCCS-Fondazione G. Pascale, 80131 Napoli, Italy; c.iannuzzi@istitutotumori.na.it (C.A.I.); m.forte@istitutotumori.na.it (I.M.F.); m.delaurentiis@istitutotumori.na.it (M.D.L.); 2 Clinical and Translational Oncology Program, Scuola Superiore Meridionale (SSM, School of Advanced Studies), University of Naples Federico II, 80131 Naples, Italy; m.tomeo@ssmeridionale.it; 3 Department of Medical Biotechnologies, University of Siena, 53100 Siena, Italy; a.sfera@student.unisi.it; 4 Dipartimento di Salute Mentale, Fisica e Medicina Preventiva, Università degli Studi della Campania “Luigi Vanvitelli”, 80122 Naples, Italy; francesco.pagano@studenti.unicampania.it; 5 Cell Biology and Biotherapy Unit, Istituto Nazionale Tumori-IRCCS-Fondazione G. Pascale, 80131 Naples, Italy; r.espositoabate@istitutotumori.na.it; 6 Sbarro Institute for Cancer Research and Molecular Medicine, Center for Biotechnology, College of Science and Technology, Temple University, Philadelphia, PA 19122, USA

**Keywords:** triple-negative breast cancer, NONO/p54, cGAS/STING, cancer therapy

## Abstract

Breast cancer (BC) remains a leading cause of cancer-related mortality worldwide, with limited treatment options for triple-negative breast cancer (TNBC). The RNA-binding protein non-POU domain-containing octamer-binding protein (NONO) has emerged as a critical regulator of tumorigenesis, but its role in immune signaling remains unexplored. We analyzed the effect of NONO protein by modulating its expression using short hairpin RNA (shRNA) and a chemical inhibitor (R)-SKBG-1. We demonstrate that NONO depletion in MDA-MB-231 TNBC cells leads to cytoplasmic DNA accumulation, micronuclei formation, and activation of the cyclic GMP-AMP synthase—stimulator of interferon genes (cGAS/STING) pathway, resulting in enhanced modulation of the immune response. NONO-deficient cells showed increased cGAS and STING activation, Tank-binding kinase 1 (TBK1) phosphorylation, nuclear factor kappa-light-chain-enhancer of activated B cells (NF-κB) nuclear localization, and transcription of pro-inflammatory genes such as CC Motif Chemokine Ligand 5 (*CCL5*). These effects were recapitulated by pharmacological inhibition using (R)-SKBG-1, confirming NONO’s immunosuppressive function. Our findings establish NONO as a key modulator of immune activation in TNBC and suggest that its inhibition may enhance anti-tumor immunity. This work paves the way for potential combination strategies involving NONO inhibitors and immune checkpoint blockade, particularly in tumors with homologous recombination deficiencies or limited immune infiltration.

## 1. Introduction

Breast cancer (BC) continues to be a major global health challenge, imposing a significant burden on healthcare systems worldwide. As the most frequently diagnosed cancer among women, it remains a leading cause of cancer-related mortality [1]. Despite significant advancements in treatment, patients with distant metastases often exhibit limited responsiveness to conventional therapies. While therapeutic targets such as Human Epidermal Growth Factor Receptor 2 (HER2), Estrogen Receptor (ER), and Progesteron Receptor (PR) have improved treatment strategies for certain BC subtypes, further research is needed to identify additional molecular players involved in cancer progression. The non-POU domain-containing octamer-binding protein (NONO) belongs to the *Drosophila* behavior/human splicing (DBHS) protein family, which interacts with DNA, RNA, and other proteins [2]. DBHS proteins share a conserved ~300-amino-acid core region, including N-terminal RNA recognition motifs (RRMs), a NOPS (NONA/ParaSpeckle) domain, and a C-terminal coiled-coil region. Due to a nuclear localization signal (NLS) at its C-terminal protein domain, NONO is predominantly found in the nucleus, mainly in paraspeckles [3]. Recent studies highlight NONO’s crucial role in tumorigenesis, influencing cell proliferation, apoptosis, migration, and DNA damage repair [4,5,6]. High NONO expression has been observed in multiple cancers, including bladder [7], lung [8,9], and others, where it is associated with increased aggressiveness. In breast cancer, NONO promotes cell proliferation by regulating the post-transcriptional expression of S-phase kinase-associated protein 2 (SKP2) and the Elongation Factor (E2F) transcription factor [10]. Moreover, NONO contributes to oncogenesis, chemotherapy resistance, and poor prognosis in triple-negative breast cancer (TNBC) by modulating Epidermal Growth Factor Receptor (EGFR) and Signal Transducer And Activator Of Transcription 3 (STAT3) stability [10,11]. Additionally, the interaction between Peptidyl-prolyl cis/trans Isomerase (PIN1) and NONO’s C-terminal Thr-Pro motifs enhances NONO stability, driving tumorigenesis by activating oncogenic genes and suppressing tumor suppressors [12]. New emerging evidence has linked cancer cells with the tumor microenvironment (TME), playing an important role in cancer development and therapy. Consistently, breast cancer shows a significant relationship with the immune compartment, which contributes to tumor initiation and progression [13]. Protein expression modulation in cancer cells can impact the generation of replication stress, leading to the accumulation of cytoplasmic DNA or DNA/RNA hybrids (R-loops) [14]. These nucleic acids are responsible for the activation of the cyclic GMP-AMP synthase - stimulator of interferon genes (cGAS/STING) pathway, regulating the immune response through the transcription of type I interferon, favoring immunogenicity in cancer cells [15]. Consistently, modulation of the BReast CAncer gene 1 (BRCA1) gene increases activation of the cGAS/STING pathway, supporting the potential development of new combination therapies comprising immune checkpoint blockade drugs for breast cancer. Given the involvement of NONO protein modulation in the regulation of replication stress and R-loops at telomeres [16], we investigated whether the NONO protein can modulate the immune response pathway as a potential new therapeutic target for cancer treatment. To this end, we altered NONO expression in MDA-MB-231 cells, a model of TNBC, using the siRNA or NONO chemical inhibitor, revealing the modulation of the cGAS/STING pathway. This study uncovered a previously unrecognized role of NONO in initiating an innate immune response, providing insights into potential therapeutic strategies for enhancing anti-tumor immunity in breast cancer.

## 2. Results

### 2.1. NONO Is Upregulated in Breast Cancer

**Figure 1:**
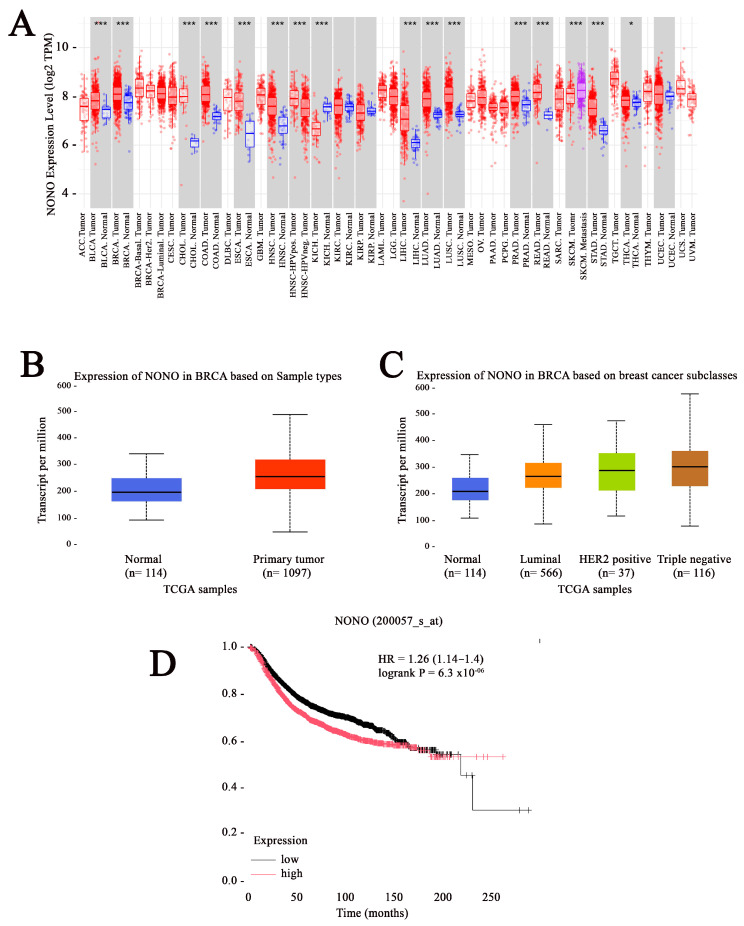
Elevated expression of non-POU domain-containing octamer-binding protein (NONO) in breast cancer. (**A**) Analysis of NONO expression across various cancer types and their corresponding normal tissues, based on data from the Tumor IMmune Estimation Resource (TIMER) database.*** *p* < 0.001 and * *p* < 0.05 (**B**) The Cancer Genome Atlas (TCGA) data accessed through University of ALabama at Birmingham CANcer data analysis Portal (UALCAN) reveal significantly increased NONO expression in breast cancer tissues compared to normal breast tissues. (**C**) NONO is markedly overexpressed across different clinical stages and molecular subtypes of breast cancer, as indicated by TCGA data from UALCAN. (**D**) Relapse-free survival analysis using KM-plotter based on NONO expression (Probe ID: 200057_s_at) in a cohort of 4929 breast cancer patients. *p*-value and hazard ratio (HR) are reported. The black and red lines represents the low and high expression NONO, respectively.

To analyze the differential expression of NONO between tumor and normal tissues, data from The Cancer Genome Atlas (TCGA) was examined using the Tumor IMmune Estimation Resource (TIMER) web tool [17]. The data from the TIMER database showed that the mRNA expression of NONO was significantly upregulated in the tumor tissues of various cancers, including breast cancer (Figure 1A). The analysis of TCGA data using The University of ALabama at Birmingham CANcer data analysis Portal (UALCAN) indicated elevated mRNA expression in breast cancer tissue compared to normal tissue and across all breast cancer subtypes, including Luminal, HER2 positive, and TNBC (Figure 1B,C) [18,19]. To determine whether the expression of NONO is associated with disease prognosis, we used a Kaplan–Meier plot (Kmplot) [20], revealing that high NONO expression was correlated with worse overall survival (OS) for all breast cancer patients (Figure 1D).

### 2.2. NONO-Deficient Cells Exhibit Increased Accumulation of Cytoplasmic DNA

**Figure 2:**
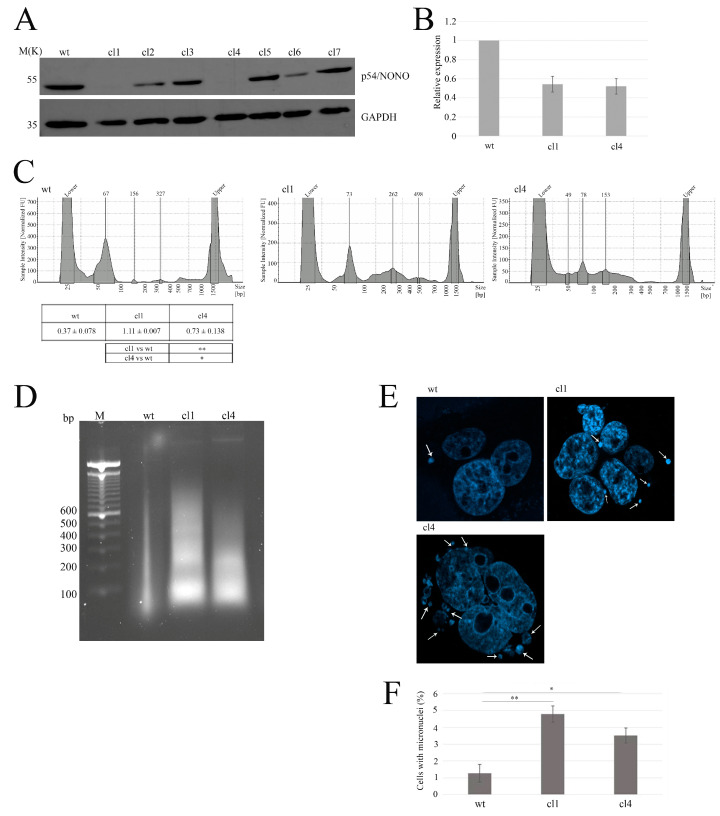
Characterization of NONO-knockout clones and associated genomic instability. (**A**) Western blot analysis of NONO protein expression in wild-type (wt) and seven knockout clones (cl1–cl7). GAPDH is shown as a loading control. (**B**) Quantitative RT-PCR analysis results showing relative NONO mRNA expression in wt, cl1, and cl4. (**C**) Assessment of cytoplasmic DNA accumulation using the Agilent Bioanalyzer, indicating increased DNA fragments in NONO-deficient clones. For cytosolic DNA analysis, protein content in the cytoplasmic fractions was first quantified to ensure equal input across samples. The table reports the means and standard deviations of at least two independent experiments. Statistical significance: ** *p* < 0.01, * *p* < 0.05. (**D**) Agarose gel electrophoresis analysis of cytoplasmic DNA isolated from MDA-MB-231 cells. DNA was extracted from the cytoplasmic fraction obtained by nuclear/cytoplasmic separation and normalized based on total protein content. Extraction was performed using the phenol–chloroform method, and samples were run on a 2% agarose gel containing ethidium bromide. The leftmost lane contains a molecular weight marker. (**E**) Representative DAPI-stained images of micronuclei in wt, cl1, and cl4 cells. White Arrows indicate micronuclei. (**F**) Micronuclei analysis of shNONO cell clones. The graph displays the mean ± SD of two independent experiments, with approximately 500 cells analyzed per condition. Statistical significance was determined using Student’s *t*-test, with results indicated as * *p* < 0.05 and ** *p* < 0.01.

First, we investigated whether NONO downregulation could increase the accumulation of nuclear DNA in the cytoplasm [21]. To achieve this aim, we generated NONO-depleted cell clones in MDA-MB-231 cells using shRNA [5]. After confirming NONO silencing using Western blotting (Figure 2A), we validated the silencing of the selected cell clones through quantitative real-time polymerase chain reaction (qRT-PCR) (Figure 2B). To assess the potential presence of nucleic acids in the cytoplasmic compartment, we performed a nuclear/cytoplasmic extraction followed by DNA purification of NONO-downregulated cells using the phenol–chloroform protocol. As illustrated in Figure 2C, shNONO cell clones showed increased cytoplasmic DNA compared to control cells, as measured using a Bioanalyzer. Consistently, the isolated cytoplasmic DNA was analyzed by agarose gel electrophoresis, confirming the previous data (Figure 2D). As reported in the literature, accumulation of cytoplasmic DNA can result from the rupture of micronuclei into the cytoplasm, which is associated with increased genomic instability [22,23,24]. We monitored the number of micronuclei in shNONO cell clones and control cells, observing an increased number in clones 1 and 4 (Figure 2E). Overall, these data reveal that NONO contributes to the increase in the cytoplasmic DNA fraction, likely resulting from micronuclei rupture.

### 2.3. Cytoplasmic DNA Activates cGAS Signaling in NONO-Depleted Cells

**Figure 3:**
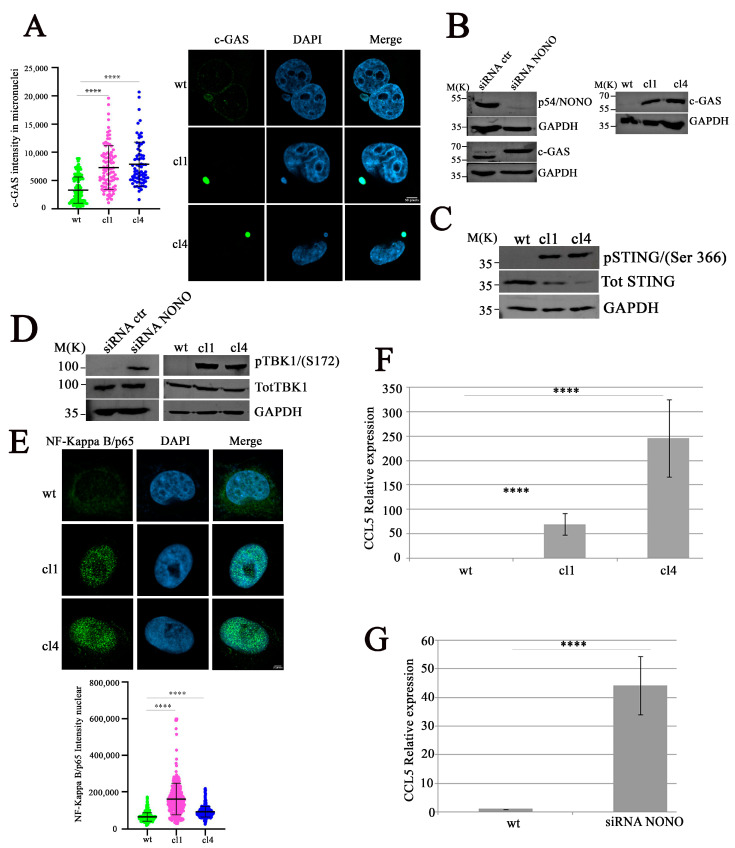
Activation of the cGAS/STING/TBK1/NF-κB pathway in NONO-knockout cells. (**A**) Quantification (left) and immunofluorescence (right) of cGAS signal intensity in micronuclei of wildtype (wt) and NONO-knockdown clones (cl1 and cl4). Images show cGAS (green), DAPI (blue), and merged channels. Knockout clones display strong cGAS localization to micronuclei. We analyzed 30 micronuclei for each condition. The results show the mean and standard deviation (SD) of three independent experiments. Statistical significance was determined using Student’s *t*-test, with results indicated as **** *p* < 0.0001. (**B**) Immunoblot analysis of NONO and cGAS proteins in MBA-MD-231 cells following siRNA transfection (left) or in shNONO cell clones (right). GAPDH is used as a loading control. (**C**) Western blot analysis of STING phosphorylation at S366 in NONO-knockdown clones compared to wt. Total STING and GAPDH are shown as loading controls. (**D**) TBK1 Ser172 was analyzed in shNONO cell clones or in transfected MDA-MB-231 cells with siNONO, followed by incubation for 72 h before Western blot analysis. Total TBK1 and GAPDH serve as controls. (**E**) Representative Images of NF-κB p65 (green), DAPI (blue), and merged channels (up panel). Quantification (down panel) of nuclear NF-κB p65 immunofluorescence in wt, cl1, and cl4 cells. The graph displays mean ± SD of two independent experiments, with approximately 450 cells analyzed per condition. Statistically significant differences are indicated by **** *p* < 0.0001. (**F**) Real-time qRT-PCR analysis of *CCL5* expression in NONO-downregulated clones compared to control cells. Results are reported as means with standard deviations of seven independent experiments. Statistical analysis was performed by subjecting the ΔCt values of treated and control samples to a two-sided Student’s *t*-test. Statistically significant differences are indicated with **** *p* < 0.0001. (**G**) MDA-MB-231 cells transfected with siRNA targeting NONO or control, followed by 72 h of incubation. *CCL5* expression in NONO-depleted cells relative to control cells was calculated using the 2^−ΔΔCt^ method. Results are presented as means ± standard deviations of six independent experiments. Statistical analysis was conducted by applying a two-tailed Student’s *t*-test to the ΔCt values of treated and control samples. Statistically significant differences are denoted by **** *p* < 0.0001.

Different studies have reported the involvement of cytoplasmic DNA in the activation of the cGAS/STING pathway through cGAS localization to micronuclei and binding to micronuclear DNA [25]. To verify whether the presence of self-DNA in the cytoplasm, mediated by NONO silencing, triggers cGAS localization in micronuclei [26], we performed confocal microscopy analysis. As illustrated in Figure 3A, shNONO clones revealed a strong enrichment of cGAS signal in micronuclei compared to controls [25]. Western blot analysis of cGAS protein levels confirmed its upregulation both in MDA-MB-231 siNONO and shNONO cell clones (Figure 3B), suggesting that the upregulation of cGAS protein is not a clone-dependent phenomenon. Upon DNA binding, cGAS produces, as a second messenger, a cyclic guanosine monophosphate-adenosine monophosphate (cGAMP), which activates the adaptor protein STING, leading to Tank-binding kinase-1 (TBK1) activation [27,28]. First, we used Western blot analysis to examine STING phosphorylation at Serine 366 (S366), which reflects its activation [29], revealing its signal in NONO-depleted cells but not in control cells (Figure 3C). We consistently demonstrated increased TBK1 phosphorylation at Serine 172 (S172) [30] in NONO-depleted cells (Figure 3D). Considering that TBK1 and its homolog IκB kinase epsilon (IKKε) can activate the IKK complex, thereby mediating the activation of the transcription factor nuclear factor κB (NF-κB) [31], we analyzed the nuclear immunofluorescence intensity of NF-κB, showing an increased signal in shNONO clone cells (Figure 3E). Overall, our data suggest that NONO modulation induces micronuclear formation, leading to the release of DNA into the cytoplasm and activation of the cGAS/STING pathway. To further investigate whether NF-κB activation contributes to the induction of immune response genes following NONO silencing, we analyzed the expression of CC Motif Chemokine Ligand 5 (*CCL5*), a well-known NF-κB target gene [32]. Using qRT-PCR, we observed a significant upregulation of *CCL5* mRNA levels in both shNONO cell clones and MDA-MB-231 siNONO-transfected cells compared to their respective controls (Figure 3F,G). These findings indicate that activation of the cGAS/STING/TBK1/NF-κB axis in the absence of NONO culminates in NF-κB-dependent transcriptional activation of pro-inflammatory genes such as *CCL5*, reinforcing the role of NONO in maintaining immune response.

### 2.4. Pharmacological Inhibition of NONO Enhances cGAS/STING-Mediated Immune Activation

**Figure 4:**
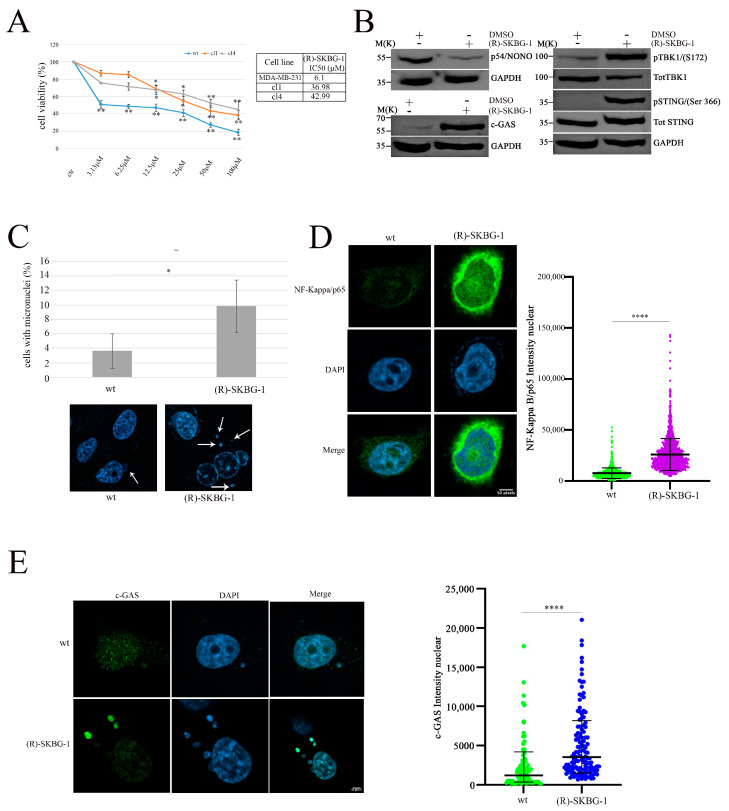
Activation of the cGAS/STING pathway and increased DNA damage in MDA-MB-231cells treated with (R)-SKGB-1. (**A**) Data represent dose–response curves reporting the mean ± standard deviation of the effects of different doses of (R)-SKGB1 on cell viability evaluated by MTS assay after 72 h of treatment in three experiments on breast cancer cell line, MDA-MB-231 wt, and shNONO cell clones. The table reports the IC50 values for each cell line. Results are expressed as a percentage of cell viability (calculated with respect to control cells treated with DMSO alone). Statistically significant differences between each dose versus control were evaluated using one-way repeated-measures ANOVA with Dunnett’s post-test and are indicated as follows: * *p* < 0.05; ** *p* < 0.01. The IC50 values were calculated using GraphPad Prism 10. (**B**) Western blot analysis showing the expression and phosphorylation levels of key proteins in the cGAS/STING pathway, including cGAS, p-TBK1, TBK1, p-STING (Ser366), and STING, in MDA-MB-231 cells treated for 48 h with (R)-SKGB-1 at its IC50 value or with DMSO as a control. GAPDH was used as a loading control. (**C**) Quantification of cells with micronuclei formation in wt and (R)-SKGB-1 cells. Representative immunofluorescence images show nuclei stained with DAPI, and white arrows indicate the micronuclei. We analyzed approximately 500 cells for each condition. The results show the mean and standard deviation (SD) of two independent experiments. Statistically significant differences are indicated by * *p* < 0.05. (**D**) Immunofluorescence staining and quantification of NF-κB p65 nuclear localization in MDA-MB-231 wt and (R)-SKGB-1-treated cells. NF-κB p65 is shown in green and nuclei in blue (DAPI). The graph displays the mean ± SD of three independent experiments, with approximately 300 cells analyzed per condition. Statistically significant differences are indicated with **** *p* < 0.0001. (**E**) Quantification of cGAS intensity in MDA-MB-231 wt and (R)-SKGB-1-treated cells. cGAS is shown in green and nuclei in blue (DAPI). We analyzed 35 micronuclei for each condition. The results show the means and standard deviations (SDs) of three independent experiments. Statistically significant differences are indicated by **** *p* < 0.0001.

To translate these findings into potential clinical applications, we employed a NONO inhibitor, (R)-SKBG-1 [33], to mimic the effects of protein expression modulation. The (R)-SKBG-1 specifically binds to cysteine 145 of the NONO protein, leading to stabilization of its complex with mRNA and inhibition of NONO protein function [33]. First, we assessed the effect of (R)-SKBG-1 on MDA-MB-231 wild-type (wt) and shNONO cell viability. Treatment with (R)-SKBG-1 exhibited cytotoxic effects after 72 h, with less toxicity in shNONO cell clones, as illustrated in Figure 4A, supporting the specificity of the (R)-SKBG-1 inhibitor. Additionally, we treated MDA-MB-231 wt cells with IC50 at 24 h (6.1 μM) of (R)-SKBG-1, followed by western blot analysis of the cGAS/STING pathway. As reported in Figure 4B, the NONO inhibitor induced upregulation of cGAS and phosphorylation of STING S366 and TBK1 S172 protein levels comparable to those observed in shNONO cell clones (Figure 3B). MDA-MB-231 wt cells treated with the NONO inhibitor revealed increased micronuclei formation together with upregulation of cGAS nuclear signal immunofluorescence (Figure 4C). Confocal microscopy analysis revealed that (R)-SKBG-1 treatment increased nuclear localization of p65 compared to control cells (Figure 4D). Finally, we observed an increase in cGAS signal in cytoplasmic micronuclei of MBA-MD-231 cells treated with (R)-SKBG-1 inhibitor (Figure 4E). Overall, these data demonstrate that treatment with the (R)-SKBG-1 NONO inhibitor confirms that NONO may be involved in the regulation of the immune response.

## 3. Discussion

Our study provides novel insights into the role of the RNA-binding protein NONO in activating immune signaling involvement through the cGAS/STING pathway. We transfected MBA-MD-231 cells with siRNA or shRNA targeting NONO mRNA, revealing an increase in cytoplasmic DNA, inducing upregulation of the cGAS/STING pathway. Modulation of the NONO protein can impact cell cycle progression [5], affecting the G1/S transition and in replication stress, potentially increasing the cytoplasmic DNA associated with the upregulation of micronuclei. Moreover, by employing NONO pharmacological inhibition with (R)-SKBG-1, we observed the modulation of the cGAS/STING pathway, as shown by the shNONO cell clones or siRNAs. These data support the role of NONO protein in preserving the immune response and the potential use of NONO protein inhibitors in cancer therapy. We modulated NONO protein activity using (R)-SKBG-1, NONO siRNA, and shNONO and observed the same biological effects on the cGAS/STING pathway. These results support the notion that off-target effects of the NONO chemical inhibitor or its silencing do not affect NONO function in the immune response. Our work contrasts with a recently published study describing the role of NONO in promoting the cGAS-mediated immune response to HIV infection [34]. The authors demonstrated that NONO is an essential sensor of the HIV capsid in the nucleus, leading to the activation of cGAS. Conversely, we showed that NONO protein downregulation in TNBC cells increases cytoplasmic DNA, leading to cGAS/STING activation. We observed increased expression of cGAS protein upon shNONO silencing or (R)-SKBG-1 treatment in MDA-MB-231 cells. In contrast, Lahaye et al. [34] reported unaffected cGAS levels upon NONO silencing in monocyte-derived dendritic cells (MDDCs). We hypothesize that the discrepancy between our data and those of Lahaye et al. is due to differences in the cell types examined. We characterized the role of NONO in immune response using MBA-MD-231 cells, TNBCs with a BRCAness phenotype [35], suggesting the possible synthetic lethal phenomenon of NONO inhibition. It will be important to determine the link between NONO downregulation and BRCA1/2 mutational status in order to define a new synthetic lethal relationship. Moreover, it may be important to test whether the downregulation of other players in the Homologous Recombination (HR), implicated in the Homologous Recombination Deficiency (HRD), could be synthetic lethal with NONO inhibition, activating the immune response. These findings highlight NONO as a potential therapeutic target for enhancing anti-tumor immunity, especially in TNBCs, where treatment options remain limited and immune checkpoint blockade (ICB) therapies have shown only partial success [36,37]. The concordance between genetic and pharmacological inhibition of NONO, in activating the cGAS/STING pathway and inducing immune-related gene expression, further supports our conclusions. Our results show that NONO-deficient cells exhibit a significant increase in cytoplasmic DNA and micronucleus formation, a hallmark of chromosomal instability. These observations are consistent with prior studies demonstrating that micronuclei serve as platforms for cGAS activation and subsequent immune signaling [38]. While we have demonstrated that NONO depletion induces immune activation in vitro, it remains crucial to confirm these findings in immunocompetent breast cancer models. Our study establishes NONO as a key modulator of the immune response activation in breast cancer. By linking NONO depletion to cGAS/STING pathway activation, we provide a new perspective on how tumor cells can be targeted to enhance anti-tumor immunity. These findings pave the way for further investigations into NONO as a therapeutic target, particularly in combination with ICB strategies. To strengthen the translational relevance of our findings, it will be essential to assess whether NONO inhibition, beyond activating the cGAS/STING pathway in vitro, can also remodel the tumor–immune interactions in vivo. In particular, NONO depletion may enhance immune cell infiltration and effector T cell activation within the tumor microenvironment, thereby sensitizing tumors to immune checkpoint blockade. This hypothesis is supported by accumulating evidence showing that cGAS/STING activation promotes type I interferon production, dendritic cell priming, and T cell recruitment, all of which are critical for effective antitumor immunity [39,40,41]. Given the limited clinical efficacy of ICB in TNBC when used as monotherapy [36,37], the combination of NONO inhibition with checkpoint inhibitors could provide a novel therapeutic avenue to overcome primary resistance. Future studies in immunocompetent breast cancer models will be crucial to validate whether NONO targeting induces a more inflamed tumor microenvironment and enhances responsiveness to immunotherapy. Such investigations could pave the way for integrating NONO inhibitors into combination immunotherapy regimens, thereby expanding the therapeutic landscape for TNBC patients. Many solid tumors develop strategies to inhibit cGAS/STING signaling, thereby reducing inflammation and immune cell infiltration [42]. NONO may be part of this immunosuppressive network by cooperating with known immune evasion factors, such as Programmed Death-Ligand 1 (PD-L1) or the Wnt/β-catenin pathway, both of which contribute to a non-inflamed tumor microenvironment [43]. Given its role in RNA metabolism, NONO might also regulate the expression of immunosuppressive cytokines such as Transforming growth factor (TGF)-β or IL-10, which further dampen T-cell activation [44]. Investigating the co-expression of NONO with these factors in patient cohorts could provide valuable insights into its relevance as a therapeutic target.

## 4. Materials and Methods

### 4.1. Cell Culture and Transfection

MDA-MB-231 (HTB-26) cells were purchased from the American Type Culture Collection (ATCC, Manassas, VA, USA) and maintained in RPMI-1640 (ThermoFisher Scientific, 11875093, Waltham, MA, USA) supplemented with 10% fetal bovine serum (FBS), 1% penicillin–streptomycin, and 1% glutamine at 37 °C in a humified atmosphere containing 5% CO_2_. All cell culture reagents were purchased from Sigma-Aldrich Corp. (St. Louis, MO, USA). To generate MDA-MB-231 clones stably expressing shRNAs, cells were seeded at 90% confluence on six-well plates and transfected with 1 μg of pSilencer5.1-shRNA through Lipofectamine 3000 (ThermoFisher Scientific, L3000008 Waltham, MA, USA). ShRNA-expressing cells were selected using 5 μg/mL of puromycin dihydrochloride (ThermoFisher Scientific, A1113803, Waltham, MA, USA), and shRNA-expressing clones were analyzed by Western blotting. To silence NONO, two 53 nt oligonucleotides (Supplementary Table S1) targeting the 732–750 region of *NONO* (NCBI NM_001145408.1) were annealed and cloned into pSilencer5.1 (Thermofisher Scientific, Waltham, MA, USA). A commercial non-targeting shRNA (shCTR) was used as a negative control. For silencing experiments, MDA-MB-231 cells were transfected with 100 nM siNONO (Supplementary Table S1) or siCTR (Horizon Discovery, D-001810-01-05, Cambridge, UK) using Dharmafect1 (Horizon Discovery, T-2001-01, Cambridge, UK) according to the manufacturer’s instructions.

### 4.2. Protein Extraction and Western Blotting

Cells were lysed on ice for 30 min in JS buffer (50 mM HEPES, pH 7.5, 1% Triton X-100, 150 mM NaCl, and 5 mM EGTA) supplemented with a PhosStop protease and phosphatase inhibitor cocktail (Merck, 4906845001, Darmstadt, Germany). Total protein content was determined by the Bradford assay (Bio-Rad, 5000001, Hercules, CA, USA). Lysates (50 µg of total proteins) were fractionated using SDS–polyacrylamide gel electrophoresis, and separated proteins were then transferred onto nitrocellulose membranes. After blocking the membranes with 5% non-fat-dried milk, they were incubated with primary antibodies against pTBK1/NAK S172 (Cell Signalling Technologies, 5483S, D52C2, Danvers, MA, USA), pSTINGS366 (Cell Signalling Technologies, 19781S, D7C3S, Danvers, MA, USA), cGAS(Cell Signalling Technologies, 15102S, D1D3G, Danvers, MA, USA), TBK1/NAK (Cell Signalling Technologies, 3504S, D1B4, Danvers, MA, USA), STING (Cell Signalling Technologies, 13647S, D2P2F, Danvers, MA, USA), p-NF-κB p65 Ser536 (Cell Signalling Technologies, 3033S, 93H1, Danvers, MA, USA), NF-κB p65 (Cell Signalling Technologies, 6956S, L8F6, Danvers, MA, USA), GAPDH (Cell Signalling Technologies, 2118S, 14C10, Danvers, MA, USA), p54nrb/NONO (Merck Millipore, 05-950, 78-1-C6, Burlington, MA, USA). After 1 h of incubation at room temperature with Goat anti-Mouse IgG (H+L) Secondary Antibody, HRP (ThermoFisher Scientific, 31430) or Goat anti-Rabbit IgG (H+L) Secondary Antibody, HRP (ThermoFisher Scientific, 31460 Waltham, MA, USA), the signals were detected with an enhanced chemiluminescence reagent (Sigma Aldrich, WBKLS0050, St. Louis, MO, USA) using an ImageQuant LAS-500 image analyzer (GE Healthcare, Chicago, IL, USA). Western blotting experiments with (R)-SKBG-1 (MedChemExpress, HY-153918, Monmouth Junction, NJ, USA) were carried out after MDA-MB-231 cells were treated, at their IC50 value, with the NONO inhibitor for 24 h.

### 4.3. Extraction and Quantification of Cytoplasmic DNA

Nuclear and cytoplasmic extraction was performed as previously described [38]. After cytoplasmic extraction, we extracted genomic DNA using the phenol–chloroform method. Briefly, cells were lysed in a lysis buffer containing Tris–HCL, pH 8.5, and an equal volume of phenol/chloroform/isoamyl alcohol (25:24:1) was added to the lysate, followed by vigorous mixing and centrifugation at high speed for 5 min to separate the aqueous and organic phases. The upper aqueous phase, containing the DNA, was carefully transferred to a new tube, and an equal volume of chloroform/isoamyl alcohol (24:1) was added. DNA was then precipitated by adding ethanol/isopropanol, followed by centrifugation. The pellet was washed with 70% ethanol, air-dried, and resuspended in water. DNA concentration and purity were assessed using a spectrophotometer. DNA derived from cytoplasmic extraction was analyzed using the TapeStation 4200 system to determine the quality and quantity between the different experimental scenarios. Briefly, 2 µL of purified DNA was combined with 2 µL of HS1000 Buffer (Agilent Technologies, Santa Clara, CA, USA) and automatically loaded onto the TapeStation 4150 platform using an HS1000 ScreenTape (Agilent Technologies). The results were analyzed using the TapeStation Analysis Software v4.1 (Agilent Technologies).

### 4.4. Cytoplasmic DNA Extraction and Agarose Gel Electrophoresis

Nuclear and cytoplasmic extraction was performed as previously described [38]. Protein concentration in the cytoplasmic extracts was quantified using the Bradford assay to ensure equal protein input across samples. DNA was then extracted from the cytoplasmic fraction using the phenol/chloroform/isoamyl alcohol (25:24:1) protocol described above. Each sample was loaded onto a 2% agarose gel containing ethidium bromide. Electrophoresis was performed in 1× TBE buffer at 100 V for 30 min. Gels were visualized under UV light, and images were acquired using an iBright 1500 (ThermoFisher Scientific, 31460 Waltham, MA, USA). A DNA ladder was included as a molecular weight reference.

### 4.5. DAPI Staining

Cells were grown on coverslips, then fixed in 4% paraformaldehyde for 15 min and permeabilized with 0.2% Triton-X 100 for 10 min. Samples were then blocked in 1% BSA for 20 min. The coverslips were mounted using the ProLong Gold Antifade Reagent with DAPI (ThermoFisher Scientific, P36930, Waltham, MA, USA). After fixation and DAPI staining, the percentage of cells with micronuclei was determined using the Zeiss LSM900 with AiryScan 2 confocal microscope equipped with a 63x/1.4 immersion oil objective. Micronuclei were defined as discrete DNA aggregates separate from the primary nucleus in cells.

### 4.6. Immunofluorescence

Cells were grown on glass coverslips, fixed with 4% paraformaldehyde in PBS for 15 min at room temperature, and washed with PBS. For cGAS staining, cells were permeabilized for 15 min with 0.25% Triton X-100 in PBS and blocked in 3% BSA for 1 h. Then, the cells were incubated with cGAS antibody (Cell Signalling Technologies, 15102S, D1D3G, Danvers, MA, USA) for 2 h at RT. The antibody was diluted in 1.5% BSA in PBS. For NF-κB p65 staining, cells were permeabilized for 15 min with 0.5% Triton X-100 in PBS and blocked in 5% BSA + 0.05% Tween20 in PBS for 1 h at RT. Then, the cells were incubated with NF-κB p65 antibody (Cell Signalling Technologies, 6956S, L8F6, Danvers, MA, USA) overnight at 4 °C. Goat anti-Rabbit IgG (H+L) Cross-Adsorbed Secondary Antibody, Alexa Fluor™ 488 (ThermoFisher Scientific, A11008, Waltham, MA, USA) was used at a 1:1000 dilution. Microscopic images were acquired using a Zeiss LSM 900 AiryScan2 with a 63x/1.4 oil objective (Zeiss, Oberkochen, Germany), and images were captured with the Zen 3.9 software (Zeiss, Oberkochen, Germany). The signal intensity of each antibody was analyzed using PRISM 9 software. The number of cells analyzed in each experiment is stated in the figure legends.

### 4.7. Real-Time Reverse Transcription (RT)-PCR

Total RNA was isolated using the RNeasy Mini Kit (Qiagen, 74104, Hilden, Germany), and 1 µg of RNA was reverse-transcribed with the iScript™ cDNA Synthesis Kit (Bio-Rad, 1708890, Hercules, CA, USA) according to the manufacturer’s recommendations. The cDNA was subjected to real-time PCR analysis using the StepOne Real-Time PCR System (Applied Biosystems, Foster City, CA, USA) with the SYBR^®^ Green Real-Time PCR Master Mix (ThermoFisher Scientific, 4309155 Waltham, MA, USA) for 40 cycles at an annealing temperature of 60 °C. Each sample was run in triplicate. The housekeeping gene *GAPDH* was used for normalization. Relative gene expression was calculated using the 2^−ΔΔCt^ method [45,46]. The sequences of the primers used are listed in Supplementary Table S1.

### 4.8. Cell Viability

(R)-SKBG-1 was dissolved in DMSO to obtain a 5 mM solution. All cells were seeded in 96-well plates at a density of 1500 cells/well (MDA-MB-231) or 2500 cells/well (NONO-depleted clones) and treated with increasing concentrations of (R)-SKBG-1 for 72 h. As a control, cells were treated with the maximum amount of DMSO used to vehicle the higher drug concentration. Cell viability was measured using the MTS assay and the CellTiter 96^®^AQueous Non-Radioactive Cell Proliferation Assay kit (Promega Corporation, G5421, Madison, WI, USA). The IC50 values were calculated using GraphPad Prism 10.

### 4.9. Statistical Analysis

Statistical analyses were conducted using GraphPad Prism (version 10 for Windows). To assess significant differences to compare all data against the control group, a one-way repeated measures ANOVA followed by Dunnett’s post hoc test was used. A *p*-value of < 0.05 was considered statistically significant. Additionally, treated and control samples were analyzed using a two-sided Student’s *t*-test. Statistically significant differences are represented as follows: * significant (*p* < 0.05) and **** very significant (*p* < 0.0001).

## 5. Conclusions

Here, we describe the role of the NONO protein in the regulation of the immune response. The modulation of NONO expression, either by shRNA or its chemical inhibitor (R)-SKBG-1, increases the activation of the cGAS/STING pathway via upregulation of cytoplasmic DNA. The modulation of the immune response through chemical inhibition of NONO by a commercially available molecule opens a potential new therapeutic opportunity for TNBC. To further validate our findings, additional studies in breast cancer models, including patient-derived organoids and syngeneic or patient-derived xenograft models, will be essential to determine the clinical relevance of NONO inhibition.

## Data Availability

Original Western blot images have been deposited at Zenodo (https://doi.org/10.5281/zenodo.15746704, accessed on 26 June 2025). The microscopy data reported in this paper will be shared by the lead contact upon request.

## Supplementary Materials

The following supporting information can be downloaded at: https://www.mdpi.com/article/10.3390/ijms26178542/s1.
