# Supplementary material for: NONO Protein Regulates the Immune Response in Human Triple-Negative Breast Cancer Cells
Source: Int J Mol Sci. 2025 Sep 2;26(17):8542. doi: 10.3390/ijms26178542 (PMC12429829; doi:10.3390/ijms26178542)
Supplement: Supplementary file 1 [file ijms-26-08542-s001.zip › ijms-3828024-supplementary.pdf]

Supplementary Materials

Table S1. List of primers.

Primer Name	Primer Sequence
sh NONO F	5′-AGAAGCTGGTTATAAAAAACCAACAATTCCACAAAGAACGAGAGCAGCCACCC-3′
sh NONO R	5′-GGGTGGCTGCTCTCGTTCTTTGTGGAATTGTTGGTTTTTTATAACCAGCTTCT-3′
siRNA-NONO	5′-GAGGUGCUAUGGGCAUAAATT-3′
NONO RT-PCR F	5′-CGCCACCACCGCCAATACC-3′
NONO RT-PCR R	5′-TCGCCTGCCTTTCCATATTTCTC-3′
CCL5 RT-PCR F	5′-GCTGTCATCCTCATTGCTACTG-3′
CCL5 RT-PCR R	5′-TGGTGTAGAAATACTCCTTGATGTC-3′
GAPDH RT-PCR F	5′-GAAGGTGAAGGTCGGAGT-3′
GAPDH RT-PCR R	5′-CATGGGTGGAATCATATTGGA-3′
siNONO	5′-GAGGUGCUAUGGGCAUAAATT-3′
